# Differences in MicroRNA Expression in Chronic Hepatitis B Patients with Early Liver Fibrosis Based on Traditional Chinese Medicine Syndromes

**DOI:** 10.1155/2020/5956940

**Published:** 2020-10-31

**Authors:** Mei-Jie Shi, Huan-Ming Xiao, Yu-Bao Xie, Jun-Min Jiang, Peng-Tao Zhao, Gao-Shu Cai, Ying-Xian Li, Sheng Li, Chao-Zhen Zhang, Min-Ling Cao, Qu-Bo Chen, Zhi-Jian Tan, Heng-Jun Gao, Xiao-Ling Chi

**Affiliations:** ^1^The Second Affiliated Hospital of Guangzhou University of Chinese Medicine, Guangdong Provincial Hospital of Chinese Medicine, Guangzhou 510120, China; ^2^Guangzhou University of Chinese Medicine, Guangzhou 510006, China; ^3^National Engineering Center for Biochip at Shanghai, Shanghai 201203, China; ^4^Institute of Digestive Diseases, School of Medicine, Tongji University, Tongji Hospital Affiliated to Tongji University, Shanghai 200065, China

## Abstract

The aim of this study was to determine if microRNA (miRNA) expression is different among chronic hepatitis B (CHB) patients with early liver fibrosis classified according to traditional Chinese medicine (TCM) syndromes. Eighteen CHB-fibrosis patients and 12 CHB patients without fibrosis were enrolled. The CHB-fibrosis group included 9 patients with the TCM syndrome of Ganyu Pixu Xueyu (GYPXXY), characterized by liver stagnation, spleen deficiency, and blood stasis, and 9 patients with the TCM syndrome of Qixu Xueyu (QXXY), characterized by deficiency of qi, blood, and blood stasis. Agilent miRNA microarray was performed first in liver specimens to determine whether miRNA expression is different in patients with these two TCM syndromes of CHB-fibrosis. Gene Ontology (GO) analysis and KEGG analysis were applied to determine the roles of the differentially expressed miRNAs. QRT-PCR was performed to validate the Agilent miRNA microarray results. Compared with GYPXXY patients, 6 differentially expressed miRNAs were upregulated (miR-144-5p, miR-18a-5p, miR-148b-3p, miR-654-3p, miR-139-3p, and miR-24-1-5p) and 1 was downregulated (miR-6834-3p) in QXXY patients. According to qRT-PCR data, miR-144-5p and miR-654-3p were confirmed as upregulated in CHB-liver fibrosis patients compared to CHB patients without fibrosis, whereas the other 4 miRNAs were not significantly different. More importantly, miR-654-3p was confirmed to be significantly upregulated in QXXY patients compared with values in GYPXXY patients, whereas no significant difference was found in miR-144-5p. Moreover, the pathways of central carbon metabolism in cancer and cell cycle related to miR-654-3p and the target genes of PTEN and ATM were found to be different between QXXY patients and GYPXXY patients. These results indicate that there are different miRNAs, pathways, and target genes between QXXY patients and GYPXXY patients. However, due to the limited sample, whether miR-654-3p and the target genes PTEN and ATM could be molecular markers to differentiate TCM syndromes could not be established.

## 1. Introduction

Chronic hepatitis B (CHB) virus infection is a major global public health problem. Twenty-five percent of patients who have the disease die from cirrhotic complications, liver failure, and hepatocellular carcinoma (HCC) [1], which is a serious risk factor. In China, more than 300,000 CHB patients die of cirrhosis and HCC every year [2]. Liver fibrosis is a precursor of HCC in CHB [3, 4]; thus, blocking the development of fibrosis or reversing it at an early stage is a worthy goal in the management of CHB.

However, antifibrotic drugs are still in the animal experimental stage, and no effective antifibrotic drug is available in Western medicine [5]. Most CHB patients with hepatic fibrosis are treated with antiviral drugs, which have the potential for promoting regression of fibrosis; however, fibrosis progresses in many CHB-fibrosis patients treated with these medications. Consequently, increasing numbers of CHB patients are using traditional Chinese medicines (TCM) for blocking liver fibrosis. Unique advantages of TCM in the treatment of fibrotic-stage liver disease without side effects have been reported [6–9]. Examples are relieving the patient symptoms, nourishing the liver, and reducing liver inflammation and fibrosis. This holistic but individualized TCM treatment can target the complex pathological mechanism of hepatic fibrosis in multiple ways.

TCM syndrome, the basis of prescription of Chinese herbs, is based on a patient's signs and symptoms, which are often subjective and not uniform. Various doctors may diagnose different TCM syndromes in the same patient [10]. Thus, it is urgent that an objective and useful method to differentiate TCM syndromes is established. Many microRNAs (miRNAs) have been correlated with liver fibrosis in CHB patients [11–13], and miRNAs have been suggested as biomarkers for liver fibrosis [14]. However, specific miRNAs have not been correlated with individual TCM syndromes. It is also unknown whether miRNAs can be used as markers for differentiation of TCM syndromes in CHB-fibrosis patients.

The TCM syndromes of Ganyu Pixu Xueyu (GYPXXY), which is characterized by liver stagnation, spleen deficiency, and blood stasis, and of Qixu Xueyu (QXXY), which is characterized by deficiency of qi, blood, and blood stasis, are the two major TCM syndromes of CHB-fibrosis at the early stage. Therefore, the current study aims to identify whether miRNAs are expressed differently between these two major TCM syndromes. Positive results will lay the foundation for the next study, which will aim to determine whether miRNAs can be a marker to differentiate TCM syndrome GYPXXY from QXXY patients among CHB-fibrosis patients.

## 2. Methods/Design

### 2.1. Patient Population and Setting

We recruited 18 CHB patients with early liver fibrosis (Scheuer fibrosis stage F1 or F2) [15] and 12 CHB patients without liver fibrosis, who were identified by screening or were enrolled in our previous study (2013ZX10005002-002). The 18 CHB patients with liver fibrosis included 9 patients with GYPXXY syndrome and 9 patients with QXXY syndrome. The current study conformed to the Declaration of Helsinki and was approved by the Ethics Committee of Guangdong Provincial Hospital of Chinese Medicine (B2013-087-01). All patients gave written informed consent before entering the study.

### 2.2. Diagnostic Criteria

Patients' diagnoses were based on the Guideline of Prevention and Treatment of Chronic Hepatitis B of Hepatology Branch of the Chinese Medical Association (2010 version) [16] and the Guidelines for Diagnostic and Treatment of Hepatic Fibrosis of the Chinese Association of Integrative Medicine [17]. The diagnoses of TCM syndromes were based on the Standards of Traditional Chinese Medicine Syndrome Differentiation for Viral Hepatitis [18] and confirmed by three senior TCM physicians, based on clinical symptoms, tongue manifestation, and pulse condition. The final result of TCM syndrome can be determined only when the results of these three experts are consistent. If the results of the three experts are inconsistent, discussion should be conducted to determine the final result.

### 2.3. Clinical and Laboratory Assessment

Demographic, clinical, and laboratory data were collected at the time of liver biopsy. Serum HBsAg was measured with electrochemical immunoassay (Elecsys 2010; Roche Diagnostics, Mannheim, Germany). Serum HBV DNA was measured with a lower limit of detection of 100 IU/mL (ABI 7300, Applied Biosystems Inc, USA).

### 2.4. Liver Specimens

All patients received liver biopsy. The liver specimens were frozen immediately in liquid nitrogen until used.

### 2.5. RNA Extraction

Total RNA was isolated by use of the TRI reagent (Merck, Darmstadt, Germany). The quantity and integrity of the extracted RNA were determined with UV spectrophotometry. Total RNA was quantified with formaldehyde denaturing gel electrophoresis.

### 2.6. Agilent miRNA Microarray

Thirty samples were analyzed with a Microarray Scanner G2505C (Agilent Technologies) using Agilent microarray slides. It contains all microRNAs from the Sanger miRBase release 22. Each glass slide is formatted with 8 high-definition 60K arrays (8 × 60K design/8 arrays with 60,000 features each). In addition to 20 replicates of each microRNA, each array carries control probes for grid alignment, as well as labeling and hybridization of control spike-ins. The Agilent microRNA Complete Labeling and Hybridization kit (Agilent Technologies) contains cyanine 3-cytidine biphosphate (pCp) for labeling; the hybridization time was 20 h at 55°C with 20 rpm. Results were analyzed with Agilent Feature Extraction software (10.7.3.1) (Agilent Technologies).

### 2.7. Data Analysis

Feature Extraction software (version10.7.1.1, Agilent Technologies) was used to analyze array images for raw data. Genespring software (version 14.8, Agilent Technologies) was used to finish the basic analysis with the raw data, which was normalized with the quantile algorithm. Probes that had at least 1 condition out of 2 conditions with flags “Detected” were chosen for further data analysis. Differentially expressed genes were identified through fold change, and a *P* value was calculated with a *t*-test. The threshold set for up- and downregulated genes was a fold change >2.0 and a *P* value <0.05. Gene Ontology (GO) analysis and KEGG analysis were, then, applied to determine the roles of the differentially expressed miRNAs. Hierarchical clustering was performed to reveal the expression pattern of distinguishable genes among samples.

### 2.8. Quantitative Real-Time PCR (qRT-PCR)

To validate the above microarray results, qRT-PCR was performed in 27 CHB patients (9 in the GYPXXY group, 6 in the QXXY group, and 12 in the nonfibrosis group). qRT-PCR was performed in a 20 *μ*l reaction mixture consisting of 2X miRcute Plus miRNA Premix (miRcute miRNA, TIANGEN, FP411-02), 0.4 *μ*m of each forward primer, and 20 ng of cDNA as the template. qPCR was performed in a Roche 480II PCR System (Roche, USA). PCR conditions were initial denaturation at 95°C for 15 min followed by 40 cycles of denaturation at 95°C for 20 s and annealing at 60°C for 34 s. After PCR amplification, melt-curve analysis of the amplicons was conducted at 60°C to 95°C, and data were collected at 0.3°C intervals.

### 2.9. Statistical Analysis

Continuous variables are expressed as the mean and standard deviation or median and interquartile range, as assessed by Student's *t*-test or a nonparametric test (Mann–Whitney), as appropriate. Categorical parameters among groups were compared with the Chi-squared test. All *P* values were two-sided, and statistical significance was set at *P* < 0.05. All statistical analyses were performed with SPSS software version 20.0 (SPSS Inc., Chicago, IL, USA).

## 3. Results

### 3.1. Clinical Characteristics of Enrolled Patients

Clinical characteristics of the three patient groups are listed in Table 1. The groups did not differ significantly in age, gender, alanine aminotransferase, and HBV DNA. There was no significant difference in the liver fibrosis stage between the GYPXXY group and the QXXY group (*P* > 0.05).

### 3.2. Agilent miRNA Microarray of Differentially Expressed miRNAs in CHB-Fibrosis Patients with Different TCM Syndromes

**Figure 1:**
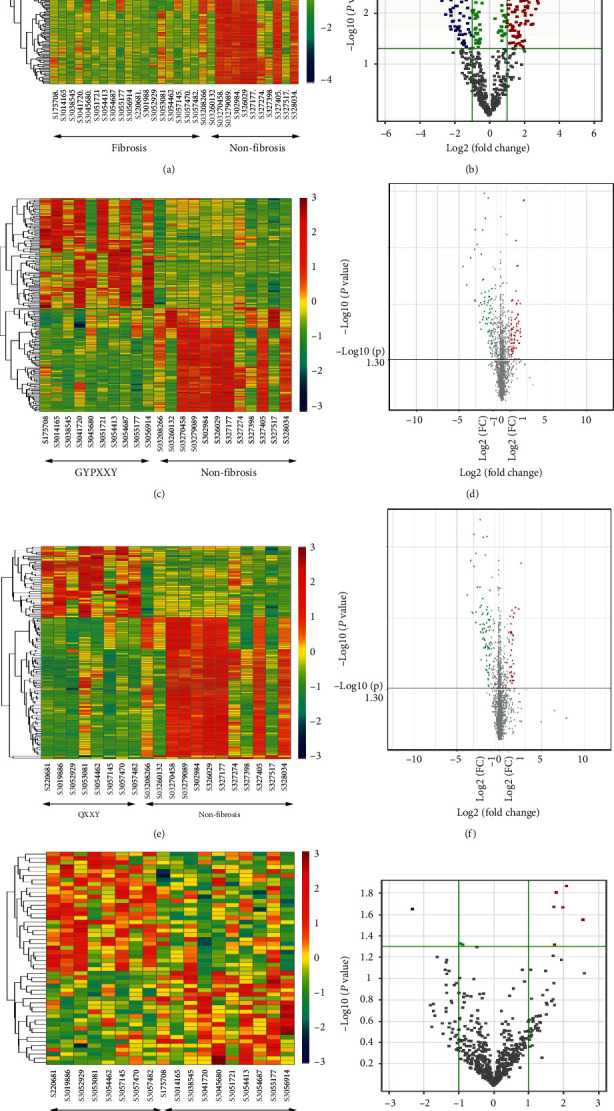
The heat maps and volcano plots of differentially expressed miRNAs among CHB-fibrosis patients with different TCM syndromes, (a) (b) differentially expressed miRNAs between the CHB patients with liver fibrosis and those without fibrosis, (c), (d) differentially expressed miRNAs between the CHB-fibrosis patients with TCM syndrome of GYPXXY and those without fibrosis, (e), (f) differentially expressed miRNAs between the CHB-fibrosis patients with TCM syndrome of QXXY and those without fibrosis, (g), (h) differentially expressed miRNAs between CHB-fibrosis patients with TCM syndrome of GYPXXY and those of QXXY.

Compared with miRNAs in CHB patients without liver fibrosis, 145 miRNAs were upregulated and 124 were downregulated in CHB patients with liver fibrosis (fold change > 2.0; *P* value < 0.05). Compared with miRNA expression in the nonfibrosis group, 155 miRNAs were differentially expressed in the GYPXXY group and 132 were differentially expressed in the QXXY group. More importantly, 7 miRNAs were differentially expressed in the QXXY group compared with expression in the GYPXXY group. Furthermore, as shown in Table 2, 6 of the 7 differentially expressed miRNAs were upregulated (miR-144-5p, miR-18a-5p, miR-148b-3p, miR-654-3p, miR-139-3p, and miR-24-1-5p), and 1 was downregulated (miR-6834-3p) in the QXXY group. The heatmap plots and volcano plots of differentially expressed miRNAs are presented in Figure 1.

### 3.3. qRT-PCR Validation of Differentially Expressed miRNAs in CHB-Fibrosis Patients with Different TCM Syndromes

**Figure 2:**
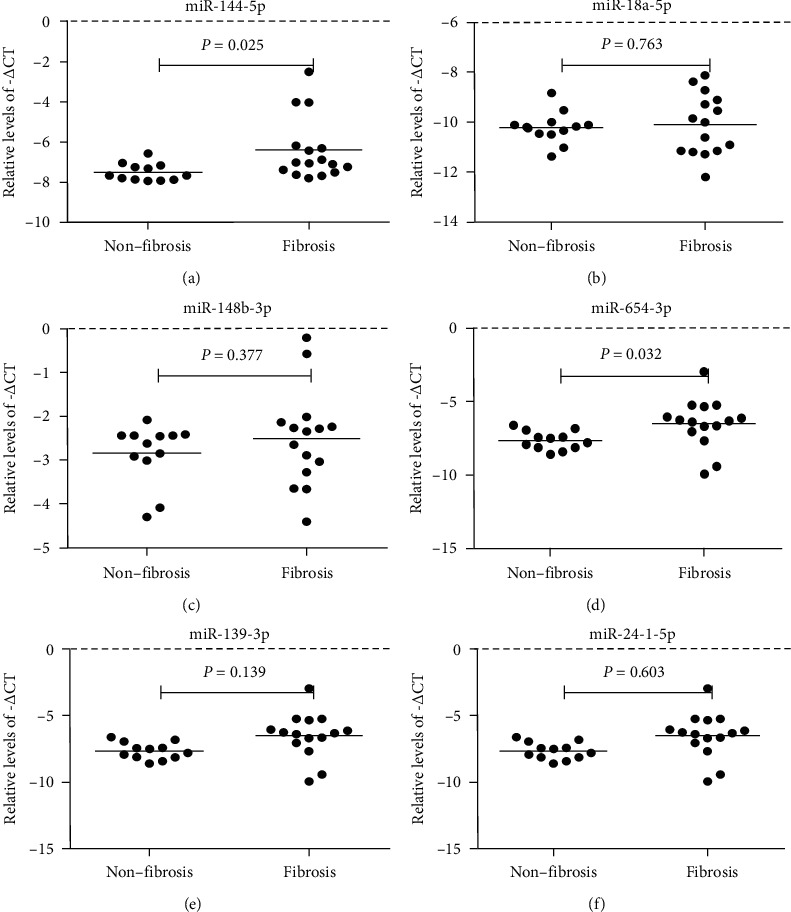
The miRNAs expressed levels from qRT-PCR data between the nonfibrosis group and fibrosis group. The levels of miR-144-5p (a), miR-18a-5p (b), miR-148b-3p (c), miR-654-3p (d), miR-139-3p (e), and miR-24-1-5p (f) in CHB patients with liver fibrosis (*n* = 15) and nonfibrosis (*n* = 12) were measured by qRT-PCR. The line at each group represents the median value of indicated miRNA.

**Figure 3:**
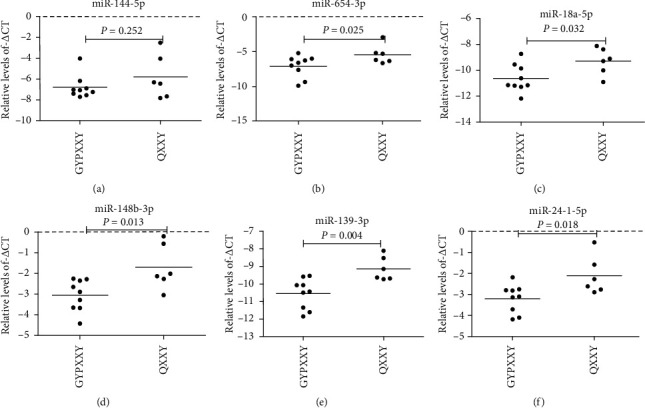
The miRNAs expressed levels from qRT-PCR data between the GYPXXY group and QXXY group. The levels of miR-144-5p (a), miR-654-3p (b), miR-18a-5p (c), miR-148b-3p (d), miR-139-3p (e), and miR-24-1-5p (f) in GYPXXY (*n* = 9) and QXXY (*n* = 6) were measured by qRT-PCR. The line at each group represents the median value of indicated miRNA.

The 7 miRNAs that were upregulated in the QXXY group, as compared to the miRNAs in the GYPXXY group, were chosen for validation by qRT-PCR in liver samples from 12 patients in the nonfibrosis group, 6 patients in the QXXY group, and 9 patients in the GYPXXY group. Six of the 7 miRNAs (miR-6834-3p excluded) were validated by qRT-PCR, and miR-144-5p and miR-654-3p were confirmed to be upregulated in CHB-liver fibrosis patients compared with values in the nonfibrosis group (Figure 2). Moreover, miR-654-3p was confirmed to be upregulated in the QXXY group compared with that in the GYPXXY group, while no significant difference was found in miR-144-5p (Figure 3). However, miR-18a-5p, miR-148b-3p, miR-139-3p, and miR-24-1-5p were not significantly different between the CHB-fibrosis group and the nonfibrosis group (Figure 2), but all these miRNAs were significantly upregulated in the QXXY group compared with values in the GYPXXY group (Figure 3).

### 3.4. miRNA Target Gene Prediction and Enrichment Analysis in CHB-Fibrosis Patients with Different TCM Syndromes

**Figure 4:**
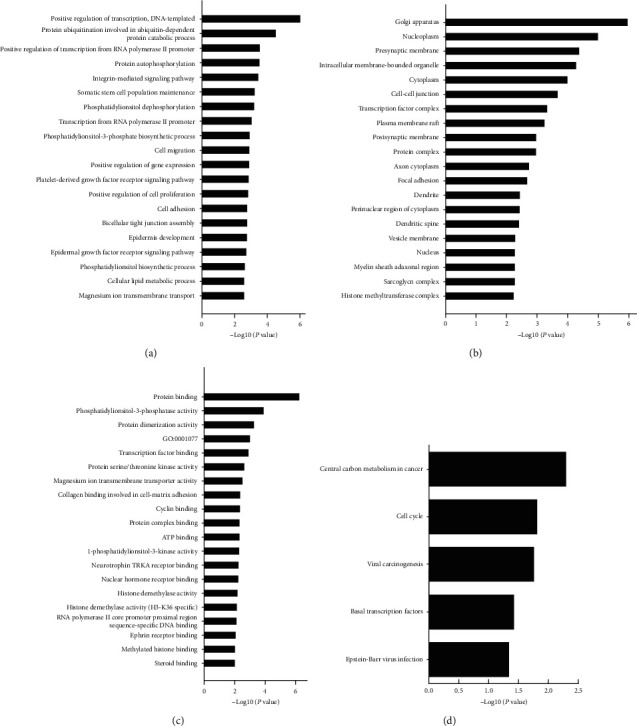
Gene Ontology and KEGG pathway analyses of differentially expressed miRNAs target genes between the GYPXXY group and QXXY group: (a) biological process, (b) cellular component, (c) molecular function, and (d) KEGG pathway analysis.

**Figure 5:**
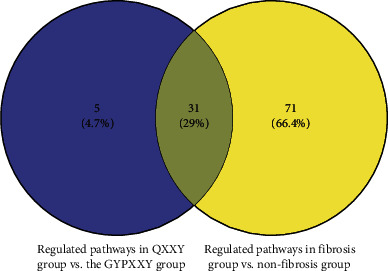
Venn analysis of differential pathways between two TCM syndromes of CHB-fibrosis patients and nonfibrosis patients.

Target scan, PITA, and miRNA org databases were used to predict the target genes of differentially expressed miRNAs. Overall, 271 common target genes from these three databases were selected for pathway analysis. The top 20 GO terms between the QXXY group and the GYPXXY group were classified by the biological process, cellular component, and molecular function (Figures 4(a)–4(c)). Positive regulation of transcription DNA-templated was the most significantly enriched biological process term. The most significantly enriched cellular component term and molecular function term were Golgi apparatus and protein binding, respectively. Further KEGG pathway analysis revealed 36 significant related pathways involved in target genes of miRNAs between the QXXY group and the GYPXXY group. Furthermore, Venn analysis was performed to identify the TCM syndrome-related pathways [19]. As a result, 31 overlapped pathways caused by disease were filtered out, and 5 differential pathways caused by different TCM syndromes were identified between the GYPXXY group and the QXXY group (Figure 5). The five pathways, as detailed in Figure 4(d), were central carbon metabolism in cancer, cell cycle, viral carcinogenesis, basal transcription factors, and Epstein–Barr virus infection. The GO terms and KEGG pathway analyses implied that molecular mechanisms are different between the two CHB-fibrosis syndromes. Moreover, three pathways, including central carbon metabolism in cancer, cell cycle, and basal transcription factors, were involved in the pathogenesis of fibrosis (Table 3). The pathways of central carbon metabolism in cancer and cell cycle related to miR-654-3p and the target genes of PTEN and ATM were different between the QXXY and GYPXXY patients.

## 4. Discussion

In the recent years, studies have demonstrated that miRNAs are potential markers for differentiation of TCM syndromes [20–23], and many miRNAs have been found upregulated or downregulated during liver fibrogenesis [13]. For example, miR-542-3p upregulation [24] and miR-454 [25] downregulation have been reported to promote liver fibrosis. However, no study has focused on miRNAs in differentiating TCM syndromes with CHB-fibrosis. This study is the first analysis of expression profiles of miRNAs in different TCM syndromes in CHB patients with fibrosis. Results of the study might provide new ideas for how to make differentiation of TCM syndromes more objective.

We used miRNA microarray to determine the expression of miRNAs in 30 samples of liver tissues of CHB patients. Six differentially expressed miRNAs were found upregulated (miR-144-5p, miR-18a-5p, miR-148b-3p, miR-654-3p, miR-139-3p, and miR-24-1-5p), whereas one was downregulated (miR-6834-3p) in the QXXY group compared with miRNA expression in the GYPXXY group. qRT-PCR analysis confirmed that miR-144-5p and miR-654-3p were upregulated in CHB patients with liver fibrosis compared with those without fibrosis. Moreover, miR-654-3p was confirmed as upregulated in the QXXY group compared with that in the GYPXXY group, while no significant difference was found in miR-144-5p. These results indicate that patients with the syndrome of QXXY might be more prone to develop liver fibrosis than patients with the syndrome of GYPXXY. Also, miR-654-3p might be a molecular marker for distinguishing TCM syndromes in CHB-fibrosis patients; we intend to explore this possibility in future studies. However, we found that miR-18a-5p, miR-148b-3p, miR-139-3p, and miR-24-1-5p did not distinguish CHB patients with fibrosis from those without fibrosis, as all these miRNAs were upregulated in the QXXY group compared with miRNAs in the GYPXXY group; this result may be due to the different miRNA levels between the QXXY group and GYPXXY group.

In this study, we also found that positive regulation of transcription DNA-templated, Golgi apparatus, and protein binding were the most significantly enriched biological process term, cellular component term, and molecular function term, respectively. Furthermore, KEGG revealed that three pathways—central carbon metabolism in cancer, cell cycle, and basal transcription factors—related to fibrosis pathogenesis were different between the QXXY group and the GYPXXY group. The GO terms and KEGG pathway analysis implied that molecular mechanisms are different between the two CHB-fibrosis syndromes. Moreover, the target genes of PTEN and ATM, as well as the differentially expressed miR-654-3p, were found related to the pathways of central carbon metabolism in cancer and cell cycle. The pathway of central carbon metabolism in cancer has been postulated to be involved in the pathogenesis of HCC [26] and liver fibrosis [27]. We found that PTEN, an important gene in this pathway, may be the target gene of upregulated differential miR-654-3p. It has been reported that upregulation of PTEN expression can inhibit the progress of liver fibrosis [28]. Therefore, we speculate that the gene PTEN has low expression in CHB-liver fibrosis associated with the TCM syndrome of QXXY compared with that of GYPXXY.

It is well known that activated hepatic stellate cells contribute to the progression of liver fibrosis. Therefore, the pathway of cell cycle, which is closely associated with the proliferation of hepatic stellate cells, may be involved in the progression of liver fibrosis [29, 30]. Interestingly, we found that ATM, an important gene in this pathway, is predicted to be the target gene of miR-654-3p. qRT-PCR data revealed that the level of miR-654-3p was significantly higher in the QXXY group than in the GYPXXY group; thus, the target gene ATM might be expressed less in the QXXY group. These results indicate that the target genes of PTEN and ATM, as well as miR-654-3p, might be important molecular markers for differentiation of the two TCM syndromes. However, this possibility requires verification in future studies.

Our study has limitations. First, due to the difficulty in obtaining liver tissues and the shortage of research funds, the number of liver samples studied for miRNAs and RT-qPCR validation was limited. Nevertheless, the number was adequate to provide proof of expression profiles of miRNAs in TCM syndromes in CHB-fibrosis patients. Second, the role of miR-654-3p in the diagnostic performances of TCM syndromes of GYPXXY and QXXY has not been assessed with the area under the receiver-operator curve, although it has been demonstrated as a potential marker for differentiating these two TCM syndromes. Third, TCM syndrome differentiation is always subjective. There is no objective method to ensure the accuracy of TCM syndrome differentiation in this study. However, we invited three senior TCM experts to determine the final result of TCM syndrome, which might partly obviate this problem.

## 5. Conclusions

Differential miRNAs were found in CHB-fibrosis patients with two common yet different TCM syndromes, GYPXXY and QXXY. miR-654-3p was more upregulated in QXXY patients than in GYPXXY patients. The pathways of central carbon metabolism in cancer, cell cycle related to miR-654-3p, and the target genes of PTEN and ATM were different between QXXY patients and GYPXXY patients. These results indicate that miR-654-3p and the target genes of PTEN and ATM might be molecular markers for differentiating the TCM of GYPXXY and QXXY syndromes, a possibility that requires additional study.

## Figures and Tables

**Table 1:** Clinical characteristics of enrolled patients.

Variables	GYPXXY group (*n* = 9)	QXXY group (*n* = 9)	Nonfibrosis group (*n* = 12)
Demographic characteristics			
Median age, years (range)	37 (25–53)	39 (26–65)	39 (24–47)
Males, *n* (%)	5 (55.6)	5 (55.6)	7 (58.3)

Laboratory data			
Median ALT, U/L (range)	24 (10–53)	23 (11–76)	18 (8–88)
Median HBV DNA (range), log10 IU/m	5.1 (2.4, 8.1)	4.8 (2.4, 8.5)	2.8 (2.4, 8.4)

Liver histology			
Inflammation activity, *n* (%)			
*G*0, *n* (%)	0	0	11 (91.7)
*G*1, *n* (%)	7 (77.8)	6 (66.7)	1 (8.3)
*G*2, *n* (%)	2 (22.2)	3 (33.3)	0

Fibrosis stage, *n* (%)			
*F*0, *n* (%)	0	0	12 (100)
*F*1, *n* (%)	5 (55.6)	4 (44.4)	0
*F*2, *n* (%)	4 (44.4)	5 (55.6)	0

^*∗*^Abbreviations: ALT, alanine aminotransferase.

**Table 2:** Differentially expressed miRNAs in CHB-fibrosis patients with GYPXXY syndrome or QXXY syndrome.

miRNA	Regulation	Fold change (QXXY/GYPXXY)	*P* values
Hsa-miR-144-5p	Up	5.93	0.028
Hsa-miR-18a-5p	Up	4.23	0.013
Hsa-miR-148b-3p	Up	3.94	0.021
Hsa-miR-654-3p	Up	3.47	0.016
Hsa-miR-139-3p	Up	3.34	0.048
Hsa-miR-24-1-5p	Up	3.32	0.021
Hsa-miR-6834-3p	Down	−4.98	0.022

^*∗*^Abbreviations: GYPXXY, TCM syndrome of Ganyu Pixu Xueyu (liver stagnation, spleen deficiency, and blood stasis); QXXY, TCM syndrome of Qixu Xueyu (deficiency of qi, blood and blood stasis).

**Table 3:** The significant pathways involved in fibrosis pathogenesis between the GYPXXY and QXXY groups after Venn analysis.

Pathways	Target genes	Differentially expressed miRNAs	*P* values
Central carbon metabolism in cancer	PTEN; KIT; HIF1A; NRAS; AKT3	Hsa-miR-18a-5p; hsa-miR-654-3p;	0.028
		Hsa-miR-148b-3p	

Cell cycle	ATM; CDC14A; CDK2; E2F3; CCND2; CDC23	Hsa-miR-18a-5p; hsa-miR-148b-3p;	0.013
		Hsa-miR-654-3p	

Basal transcription factors	GTF2H1; TBPL1; TAF4B	Hsa-miR-18a-5p; hsa-miR-148b-3p	0.021

^*∗*^Abbreviations: GYPXXY, TCM syndrome of Ganyu Pixu Xueyu (liver stagnation, spleen deficiency and blood stasis); QXXY, TCM syndrome of Qixu Xueyu (deficiency of qi, blood and blood stasis).

## Data Availability

The datasets supporting the conclusions of the current study are available in the Guangdong Provincial Hospital of Chinese Medicine.
